# *Prakriti* (Ayurvedic concept of constitution) and variations in platelet aggregation

**DOI:** 10.1186/1472-6882-12-248

**Published:** 2012-12-10

**Authors:** Supriya Bhalerao, Tejashree Deshpande, Urmila Thatte

**Affiliations:** 1 Dept. of Clinical Pharmacology, TN Medical College & BYL Nair Ch. Hospital, Mumbai Central, Mumbai, 400 008, India; 2 Dept. of Clinical Pharmacology, Seth GS Medical College and KEM Hospital, Parel, Mumbai, 400 012, India

**Keywords:** Adenosine diphosphate, Aspirin, *Pitta*, *Kapha*, *Vata*

## Abstract

**Background:**

Ayurveda, the Indian traditional system of medicine describes a unique concept “*prakriti*”, genetically determined, categorising the population into several subgroups based on phenotypic characters like appearance, temperament and habits. The concept is claimed to be useful in predicting an individual’s susceptibility to a particular disease, prognosis of that illness and selection of therapy. The present study was carried out to study if the platelet aggregatory response and its inhibition by aspirin varied in the different *prakriti* subtypes.

**Methods:**

After obtaining Institutional Ethics Committee permission, normal healthy individuals of either sex between the age group 18 to 30 years were recruited in the study. Their p*rakriti* evaluation was done using a standardized validated questionnaire (TNMC *Prakriti* 2004). Their Platelet Rich Plasma was incubated with either aspirin [2.5micro-mole (μM) and 5μM] or distilled water as control for three minutes after which the aggregatory response to 5μM Adenosine Diphosphate (ADP) was measured over a period of 7 minutes.

**Results:**

We observed that in the study population of normal healthy participants (n= 137), ADP-induced maximal platelet aggregation (MPA) was highest among the *Vata-pitta prakriti* individuals [Median (range), 83.33% (52.33-96)] as compared to the other *prakriti* types and these individuals responded better to lower dose of aspirin compared to other *prakriti* types.

**Conclusions:**

Our results suggest that identifying the *prakriti* may help in individualising therapy or predicting proneness to a disease.

## Background

Ayurveda, the Indian traditional system of medicine [1] describes a unique concept “*prakriti*” [2] (constitution), which is genetically determined, categorising the population into several subgroups based on phenotypic characters like appearance, temperament and habits. The concept is claimed to be useful in predicting an individual’s susceptibility to a particular disease, prognosis of that illness and selection of therapy [3].

Ayurveda attributes these constitutional characteristics of an individual to the preponderance of certain “*doshas*”. Three main *doshas* are described, *viz. vata, pitta* and *kapha*. *Kapha dosha* is the “anabolic”, synthetic *dosha*, responsible for growth and maintenance of structure [4]. The *pitta dosha* is the one responsible for metabolism, including digestion in the gut, and cellular or sub-cellular metabolism. *Vata dosha* is responsible for movement (muscular, nervous energy etc.). Based on the predominance of individual *doshas,* there are three major types of *prakriti* named after predominant *dosha*, *viz., vata*, *pitta* and *kapha*. The *prakriti* is believed to be determined at the time of conception and is influenced by the *milieu interior* of the womb and the dietary habits and lifestyle of the mother [5]. These *prakriti*s exhibit attributes of the dominant *Dosha* in physical, physiological and psychological characteristics. The disturbance in equilibrium of these *doshas* can lead to disease according to the *prakriti* of the person for example; a *pitta prakriti* person is described to be more prone to peptic ulcers, hypertension, and skin diseases, a *vata prakriti* person to backache, joint aches and crackling joints while individuals with *kapha prakriti* are prone to obesity, diabetes and atherosclerosis [6-8].

Various studies have tried to establish whether specific *prakriti* individuals show specific groupings according to anthropometric measurements and biochemical variables like serum cholesterol, blood sugar or blood groups [9]. Recently a study reported complete absence of the HLA DRB1*02 allele in the *Vata *type and of HLA DRB1*13 in the *Kapha* type. Higher allele frequency of HLA DRB1*10 was noted in the *Kapha* type than in the *Pitta* and *Vata* types [10].

In another study, the differences in the whole gene expression among the various *prakriti* subtypes have also been explored. The functional categories of genes showing differential expression among *Prakriti* types were significantly enriched in core biological processes like transport, regulation of cyclin dependent protein kinase activity, immune response and regulation of blood coagulation. A significant enrichment of housekeeping, disease related and hub genes were also observed in the three extreme constitution types [2].

In our earlier studies we found a specific type of *prakriti* was predominant in epileptic or hypertensive individuals as compared to normal healthy individuals [7]. Platelet reactivity, the phenomenon that markedly influences the pathological outcome of thrombosis and sensitivity to anti-platelet drugs, has been reported to exhibit individual/genetic variations [11]. Since *prakriti* has been described to have genetic origin in Ayurvedic texts [5] we thought it would be interesting to study whether platelet aggregation in response to ADP and its inhibition with aspirin varies in the different *prakriti* sub-types.

## Methods

### Ethics

The study was initiated after obtaining permission from Institutional Ethics Committee, BYL Nair Ch. Hospital & TN Medical College, Mumbai and conducted according to the principles enunciated in the Declaration of Helsinki (2008) [available at http://www.wma.net/en/30publications/10policies/b3/index.html last accessed on 8^th^ June 2012] and the ICMR’s Ethical Guidelines for Biomedical Research in Human Participants [available at http://icmr.nic.in/ethical_guidelines.pdf last accessed on 8^th^ June 2012]. Only those participants who gave written informed consent were recruited.

### Study population

Normal healthy individuals (confirmed by history, physical examination and routine laboratory investigations, including hematology, renal and liver function tests) between the age group of 18 to 30 years (both years inclusive) of either sex, and willing to abide by trial procedures were enrolled.

### ***Prakriti*** assessment

*Prakriti* was assessed using a multiple-choice questionnaire (Additional file 1: TNMC *Prakriti* 2004) which was designed on the basis of literature in Ayurvedic texts comprising 37 objective questions related to the person’s physical characteristics, psychological make-up and physiological habits (Table 1). Each of the questions had three options to choose from referring to a property attributed to *Vata* (V)*, Pitta* (P) or *Kapha* (K). The score obtained by a person for answers in the V, P and K domain were summed up and the person was identified as having a specific *prakriti* depending on scores obtained. When a participant scored ≥ 50% on a particular *dosha,* that was considered as the predominant *dosha*, whereas a score between 25%- 35% categorised the *dosha* as the secondary *dosha* in the *prakriti*. For example, when a participant scored 20 (54%) for the *Pitta dosha* and 13 (32%) for the *Kapha dosha,* he was categorised as a *Pitta-Kapha prakriti*. On the other hand if the score was 22 (59%) for the *Kapha dosha* and 12 (32%) for the *Pitta dosha,* he was categorised as *Kapha-Pitta prakriti*.

**Table 1:** Demographic details (n=137)

***Prakriti***	**n (M:F)**	**Age (years)**	**BMI (kg/m**^**2**^**)**
*Vata-Kapha*	4 (1:3)	23 ± 1.22	18.99 ± 2.57
*Vata-Pitta*	16 (7:9)	22.94 ± 2.08	19.71± 2.47*
*Pitta-Kapha*	65 (32:33)	23.08 ± 1.71	21.81 ± 2.67
*Pitta-Vata*	12 (7:5)	23.25 ± 2.45	20.37 ± 2.79
*Kapha-Vata*	4 (1:3)	23.50 ± 2.08	20.34 ± 2.21
*Kapha-Pitta*	36 (19:17)	23.14 ± 2.28	23.42 ± 3.89

*p<0.05 as compared to *Kapha-Pitta* using One-way ANOVA.

### Validation of questionnaire

The questionnaire was validated by pre-testing where the results obtained by the questionnaire were confirmed by the clinical assessment of the *prakriti* independently by two Ayurveda physicians in 30 participants. More than 90% concordance was observed in the *prakriti* assessment by the two clinicians as well as by the questionnaire.

### Evaluation of ***prakriti*** in participants

For the study, the *prakriti* of each volunteer was assessed using the validated questionnaire (Additional file 1: TNMC *Prakriti* 2004). It was further confirmed by an Ayurvedic physician (first author) following a traditional algorithm (based on interview and a physical examination) to assess various physical, physiological and psychological characters as described in Ayurvedic texts [5].

The individual was classified into *vata-pitta, vata-kapha, pitta-kapha, pitta-vata, kapha-pitta* or *kapha-vata prakriti*.

### Platelet aggregation study

Platelet aggregatory response to ADP was studied using the turbidometric method described by Born [12] on a Chronolog platelet aggregocorder. Briefly, 9ml blood was collected from each volunteer in a polystyrene tube containing 1 ml of 3.8% sodium citrate. Platelet rich plasma (PRP) was obtained as a supernatant by centrifugation (1000 rpm for 10 mins at 25°C) and the remaining blood was centrifuged again (4000 rpm for 15 mins at 25°C) to obtain platelet poor plasma (PPP). Platelets in the PRP were counted using a platelet counter and the count was adjusted to 2 × 10^5^/L using autologus PPP for dilution. This plasma was incubated with either aspirin (2.5μM and 5μM) or distilled water as control for three minutes after which the aggregatory response to 5μM Adenosine Diphosphate (ADP) was measured over a period of 7 minutes. The percent aggregation at this time point was defined as the Maximal Platelet Aggregation (MPA). dMPA was calculated by subtracting % MPA with aspirin from % MPA with distilled water.

### Reproducibility of platelet aggregation studies

Platelet aggregation studies were repeated thrice at an interval of 15 days in the initial 76 participants to identify whether there was variability in platelet aggregation over a period of time. Results suggested that there was no significant variation in platelet function over time and hence platelet aggregation was studied only once in the rest of the participants (n=61). The average of the three readings of the initial 76 participants was used for analysis.

### Statistical analysis

In the absence of any preliminary data, no formal sample size calculation was performed.

The data was expressed as Mean ± SD, 95% CI if distributed normally and was analysed using One-way ANOVA followed by Tukey’s post-hoc test. The data was expressed as Median (Range) if not normally distributed and was analysed using Kruskal Wallis followed by Dunn’s post-hoc test. Graphpad Instat software, version 3.06 was used for all analyses. The level of significance for all analysis was taken as p<0.05. Two *prakriti* types viz. *Vata-kapha* and *kapha-vata* could not be considered for statistical analysis because of too few values in these groups.

## Results

A total of 137 participants were recruited in the study. The distribution of these volunteers according to *Prakriti* along with their demographic data is shown in Table 1. The age and sex distribution of the participants amongst the 3 *prakrti* types was comparable. Interestingly, the BMI in *vata-pitta prakriti* was significantly less as compared to *kapha-pitta prakriti.*

Although the *vata-pitta prakriti* individuals had the maximum MPA as compared to other *prakriti* individuals (Table 2) this was not statistically significant. After incubation with 2.5 or 5μM aspirin, % MPA in all *prakriti* types was comparable.

**Table 2:** ADP (5μM) induced % Maximal platelet aggregation (%MPA) (n=137) with or without aspirin [Median (Range)]

***Prakriti***	**Distilled water**	**With Aspirin 2.5μM**	**With Aspirin 5μM**
*Vata-Kapha*	86 (57-93)	52 (44-59)	55.33 (28-70)
*Vata-Pitta*	83.33 (52.33-96)	49 (32-91)	51.50 (25.67-79)
*Pitta-Kapha*	78 (20-115.33)	58.17 (14-88.33)	48 (5-78)
*Pitta-Vata*	69.50 (56.33-106)	50.50 (29-84)	44 (20-60)
*Kapha-Vata*	77.50 (69-82)	51.50 (44-57)	41.17 (38-48)
*Kapha-Pitta*	72 (35.67-98)	51.67 (22-86)	40 (14.67-68)

The largest change (dMPA) in the MPA after aspirin was noted in the *vata-pitta prakriti* individuals followed by those with *kapha-vata* and *pitta-vata prakriti*. Individuals with *pitta-kapha* and *kapha-prakriti* showed the least inhibition of platelets at 2.5μM concentration. This was significantly less than that in individuals with *vata-pitta prakriti* (p<0.05). However, at 5μM aspirin, no difference was observed amongst *prakriti* types suggesting that a plateau effect was reached with aspirin (Table 3).

**Table 3:** **dMPA according to *prakriti *(n=137) [Median (Range)]**

***Prakriti***	**Concentrations of Aspirin**	
	**2.5μM**	**5μM**
*Vata-Kapha*	29 (13-44)	29.17 (23-32)
*Vata-Pitta*	31.83 (3-57)	27.83 (11-51)
*Pitta-Kapha*	18 (3-48)*	27.33 (14-64)
*Pitta-Vata*	19.33 (12-40)	29.5 (21.33-49)
*Kapha-Vata*	22.67 (18.67-38)	32.83 (28-44)
*Kapha-Pitta*	16.67 (6-60)*	27.33 (9-71)

*p<0.05 as compared to *vata-pitta prakriti* using Kruskal-Wallis test followed by Dunn’s post-test.

## Discussion

We found in our cohort of normal healthy individuals that platelet aggregation induced by ADP and the inhibitory effect of aspirin differed according to *prakriti*. Thus, *vata-pitta prakriti* individuals had the maximum MPA as compared to other *prakriti* types. These individuals also demonstrated largest dMPA after exposure to 2.5μM aspirin.

According to Ayurveda, *Vata dosha* is reported to be responsible for a quick response or rapid movement, whereas *Pitta dosha* is described as the *dosha* responsible for metabolic activities (platelet aggregation occurs secondary to tremendous enzymatic activity within and around the platelets). These properties of *doshas* probably are also reflected in cellular responses. In our study too the *vata-pitta prakriti* individuals had the maximum platelet aggregation.

Our data further shows that presence of *kapha dosha* lowers the aggregatory response. According to Ayurveda, *Kapha dosha* leads to slow metabolism [5] which may be reflected as a slower platelet aggregatory response.

Interestingly, our study highlights the subjective nature of *prakriti* analysis indicating it is difficult to quantify the influence of each *dosha* on the platelet response objectively e.g. *pitta-kapha prakriti* participants had a median MPA of 78 with a range of as low as 20 (perhaps this participant had a higher *kapha* influence) to as high as 115 (perhaps having higher *pitta* influence), while individuals with the *kapha-pitta prakriti* (who have combination of the same 2 *doshas* in different proportions) had a median MPA of 72 with a range of only 35.67 to 98 suggesting that the dominant *kapha dosha* in these participants tended to keep the aggregatory response low on the whole. These findings suggest that the predominant *dosha* determining *prakriti* might be playing major role in the process of platelet aggregation. However, these findings need to be confirmed in a larger sample size with thorough quantitative analysis of *prakriti* determining *doshas.*

It is also important to note that ADP induced platelet aggregation is a complex process *in vivo*, influenced by various factors including levels of endogenous ADP, cGMP and Arachidonic acid, blood viscosity [13] and family history [14]. Therefore, the results of our study may not reflect the *in vivo* situation.

ADP is known to be central amongst agonists of platelet aggregation that act upon surface expressed G protein-coupled receptors P2Y_1_ and P2Y_12_. A recent study [15] has demonstrated that P2Y_12_ H2 haplotype is associated with the maximal aggregation response to ADP [14]. Intra-individual differences in anti-platelet effect of aspirin have also been reported and association of these differences with polymorphisms in the genes coding for cyclo-oxygenase-1 (COX-1) and several platelet glycoprotein (GP) receptors have also been studied [16].

Patwardhan *et al *[17] have shown that the extensive metabolizer (EM) genotype of the drug metabolizing enzyme CYP2C19 (*1/*3) was found only in *Pitta Prakriti*. The higher metabolic capacity of the *pitta prakriti* is similar to the higher platelet aggregation associated with *pitta* in our study. It would be therefore interesting to study whether the differences we observed in the present study are due to the genetic variations amongst *prakriti* types*.*

A possible limitation of our study is unequal number of individuals in different *prakriti* types and the small sample size (n=4) in *vata-kapha* and *kapha-vata* types, Further, there is evidence that the inter-individual differences in platelet aggregatory response to ADP are more pronounced at lower concentrations of ADP (1, 2 or 5 μM) [15]. Hence, to detect the variations in %MPA amongst *prakriti* subtypes, study of platelet aggregation using 1 and 2 μM ADP may prove more useful.

A recent study has shown that there is association between blood group of individual and risk of heart disease [18]. Although we have not done blood grouping of volunteers recruited in our study, it would be worth exploring whether blood groups have any association with *Prakriti* as well. This association can prove an important bridge between Ayurvedic and modern medical concepts.

The findings of our study however can have implications with respect to pharmacogenomics & study of dose-response relationships. These findings can also prove useful for the randomization in clinical trial design, as randomization would be best within the specific *prakriti* or *dosha* predominan*t* sub-groups than across an aggregated population.

## Conclusions

Our study for the first time documents *prakriti* related variations in platelet aggregation response in healthy individuals.

## Abbreviation

Nil: (explained in text).

## Competing interests

Authors declare that there is no competing financial interest in relation to the work described.

## Authors’ contributions

SB: Design of the study, analysis and interpretation of data, drafting the manuscript. TD: Acquisition of data. UT: Conception and design, interpretation of data, revising the manuscript critically for intellectual content. All authors read and approved the final manuscript.

## Pre-publication history

The pre-publication history for this paper can be accessed here:

http://www.biomedcentral.com/1472-6882/12/248/prepub

## Supplementary Material

Additional file 1 TNMC Prakriti 2004 Questionnaire. Click here for file
