# Supplementary material for: Prakriti (Ayurvedic concept of constitution) and variations in platelet aggregation
Source: BMC Complement Altern Med. 2012 Dec 10;12:248. doi: 10.1186/1472-6882-12-248 (PMC3562518; doi:10.1186/1472-6882-12-248)
Supplement: Additional file 1 — TNMC Prakriti 2004 Questionnaire. [file 1472-6882-12-248-S1.doc]

TNMC Prakriti 2004 Questionnaire

Date: *Prakriti*:

Name: Age: Sex:

Birth date: Actual birthplace: Place of intra-uterine life

Height: _____ cms. Weight: _____kg. Body Mass Index: ______

Occupation: _______________

Address: ________________________________

|  | *Vata* | *Pitta* | *Kapha* |
| --- | --- | --- | --- |
| Total points |  |  |  |

| **No.** | **Character** | ***Vata*** | ***Pitta*** | ***Kapha*** |
| --- | --- | --- | --- | --- |
| 1.  2. | Body frame  Body Mass Index | Lean long  < 19 | Medium  19-25 | Large, plump, fleshy, fatty  > 25 |
| 3.  4.  5. | **Speech**  Speed  Clarity  Character | Fast  Diffuse words  Easily deviates from the topic, more talkative | Fast  Clear  Impressive speaker | Slow  Clear  Less talkative, likes to be reserved |
| 6. | **Eyes**  Colour- Sclera | Blackish | Reddish, brown | Milky white  Edges- reddish |
| 7.  8. | **Lips**  Character  Colour | Cracked, shapeless  Blackish | Smooth, soft, thin  Reddish | Smooth, glossy, Proportionate  Pinkish |
| 9.  10. | **Nails**  Character  Colour | Small, Cracking, breaking, rough, easily break  Blackish | Small, smooth &flat  Reddish | Big, smooth, glossy  Pinkish |
|  |  |  |  |  |

| 11.  12.  13. | **Hair**  Texture  Colour  Thickness | Rough & Dry  Black  Less | Soft & Delicate  Gray/ Brown  Medium | Soft & Shiny  Black  More |
| --- | --- | --- | --- | --- |
| 14.  15.  16. | **Skin**  Character  Colour  Temperature | Cracking, rough  Blackish tinge  Cold | Soft, oily, with moles, pimples, freckles  Yellowish tinge  Warm | Smooth, glossy  Fair, pinkish  Cold |
| 17. | **Body odor** | Absent | Present | Absent |
| 18.  19.  20.  21. | **Appetite**  Frequency of eating  Quantity at meal  Habit  If meal is skipped/ meal timings are changed/ style of food is changed | More  Less  Irregular  Constipation | More  More  Profound  Headache/vomiting  Headache, vomiting | Less  More  Not much  Nothing special |
| 22. | **Thirst** | Irregular | More | Less |
| 23.  24.  25. | **Stool**  Habit  Consistency  Colour | Irregular  Hard  Blackish | Regular  Semi-solid  Yellowish | Regular  Well-formed  Yellowish |
| 26.  27. | **Sleep**  Character  Duration | Interrupted, less  6 hours | Uninterrupted, less  6-8 hours | Sound, profound  8 hours or more than 8 hours |
| 28. | **Excitement** | Quickly, cools down quickly | Quickly, does not cool down quickly | Rarely |
| 29. | **Working style** | Quickly | Medium | Slowly |
| 30. | **Other movements** | Fast, unnecessary | Fast, precise | Slow steady |

| 31. | **Strength** | Less, feel exhausted after doing some work | Medium, moderately gets tired | Good, do not feel tired |
| --- | --- | --- | --- | --- |
| 32. | **Style of tackling problem** | Worrying continuously without expressing | Losing self control, becoming angry/ irritated | With cool and stable mind |
| 33. | **Control on desires** | Hardly, doesn’t work hard for the same | Cannot, work hard, achieve it | Can control easily |
| 34. | **Concentration on work** | Lack of concentration | Can concentrate on thing of interest | Can easily concentrate |
| 35.  36.  37. | **Cognition Process**  Grasping  Store  Memory | Quick, poor  Poor  Less | Quick, good  Average  Average | Delayed  Good  Good |
